# Shared Genetics and Couple-Associated Environment Are Major Contributors to the Risk of Both Clinical and Self-Declared Depression

**DOI:** 10.1016/j.ebiom.2016.11.003

**Published:** 2016-11-04

**Authors:** Yanni Zeng, Pau Navarro, Charley Xia, Carmen Amador, Ana M. Fernandez-Pujals, Pippa A. Thomson, Archie Campbell, Reka Nagy, Toni-Kim Clarke, Jonathan D. Hafferty, Blair H. Smith, Lynne J. Hocking, Sandosh Padmanabhan, Caroline Hayward, Donald J. MacIntyre, David J Porteous, Chris S. Haley, Andrew M. McIntosh

**Affiliations:** a Division of Psychiatry, University of Edinburgh, Edinburgh, UK; b MRC Human Genetics Unit, Institute of Genetics and Molecular Medicine, University of Edinburgh, Edinburgh, UK; c Generation Scotland, Centre for Genomic and Experimental Medicine, Institute of Genetics and Molecular Medicine, University of Edinburgh, Edinburgh EH4 2XU, UK; d Centre for Cognitive Ageing and Cognitive Epidemiology, University of Edinburgh, Edinburgh, UK; e Medical Genetics Section, Centre for Genomic and Experimental Medicine, Institute of Genetics and Molecular Medicine, University of Edinburgh, Edinburgh, UK; f Division of Population Health Sciences, University of Dundee, Dundee, UK; g Division of Applied Health Sciences, University of Aberdeen, Aberdeen, UK; h Institute of Cardiovascular and Medical Sciences, University of Glasgow, Glasgow, UK; i The Roslin Institute and Royal (Dick) School of Veterinary Sciences, University of Edinburgh, UK

**Keywords:** Major depressive disorder, Self-declared depression, SNP heritability, Couple effect, Family environment, Linear mixed modeling

## Abstract

**Background:**

Both genetic and environmental factors contribute to risk of depression, but estimates of their relative contributions are limited. Commonalities between clinically-assessed major depressive disorder (MDD) and self-declared depression (SDD) are also unclear.

**Methods:**

Using data from a large Scottish family-based cohort (GS:SFHS, N = 19,994), we estimated the genetic and environmental variance components for MDD and SDD. The components representing the genetic effect associated with genome-wide common genetic variants (SNP heritability), the additional pedigree-associated genetic effect and non-genetic effects associated with common environments were estimated in a linear mixed model (LMM).

**Findings:**

Both MDD and SDD had significant contributions from components representing the effect from common genetic variants, the additional genetic effect associated with the pedigree and the common environmental effect shared by couples. The estimate of correlation between SDD and MDD was high (r = 1.00, se = 0.20) for common-variant-associated genetic effect and lower for the additional genetic effect from the pedigree (r = 0.57, se = 0.08) and the couple-shared environmental effect (r = 0.53, se = 0.22).

**Interpretation:**

Both genetics and couple-shared environmental effects were major factors influencing liability to depression. SDD may provide a scalable alternative to MDD in studies seeking to identify common risk variants. Rarer variants and environmental effects may however differ substantially according to different definitions of depression.

## Highlights

• Shared genetics and couple-associated environment are major contributors to the risk of depression.
• The common-variant-associated genetic correlation between MDD and SDD was very high (r = 1.00, se = 0.20).
• Lower correlations were detected for pedigree-associated genetics and couple-shared environmental components.

It is important to understand the differences between clinical depression (MDD) and self-declared depression (SDD) for which there has been recent genetics progress. We found major contributions from genetics and couple-shared environment to both traits. There is a very high correlation between the traits associated with common genetic variants but a lower correlation for other (probably rarer) genetic variation. MDD and SDD also likely differ in their shared environmental risk factors. Thus for studies of common genetic variation, SDD is a potential alternative to MDD. In clinical practice and research, the spousal depression status should also be considered a risk indicator.

## 1 Introduction

Depression has a pattern of familial aggregation, which implies the influence of genetic effects, common environmental effects shared by relatives, or both. The genetic component (heritability) has been estimated by a twin study of major depressive disorder (MDD) to be 37% (Sullivan et al., 2000). The SNP heritability (heritability attributed to common genetic variants) of MDD varies across populations and samples (21%–32%) (Lubke et al., 2012, Lee et al., 2013). Subsequently, a ‘children of twins’ study found a significantly greater risk of depression in children of depressed monozygotic (MZ) twins than in the offspring of their non-depressed twin. This implies a potential environmental effect of parental depression on offspring (Singh et al., 2011). Studies have also shown that having a partner with psychiatric disorder may increase an individual's risk of MDD (Joutsenniemi et al., 2011, Desai et al., 2012), but meta-analytic studies suggest no effect of the shared sibling environment and other studies have postulated more complex relationships (Olino et al., 2006). Whilst each of these studies separately provided evidence for the genetic and familial environmental components in depression, a precise separation of these potential effects should involve estimating them simultaneously in the same model and has yet to be achieved.

The accurate separation and estimation of the genetic and environment components on liability to depression provide crucial information, as it reveals the upper limit of the genetic effects, the probability of true positive results from genetic studies and the potential for accurate risk predictions for depression (Makowsky et al., 2011, Tenesa and Haley, 2013). Genetic studies attempting to map causal variants have been performed for various definitions of depression. These include clinically-assessed depression, self-report of clinical diagnosis of depression and self-reported depressive symptoms (consortium, C, 2015, Major Depressive Disorder Working Group of the Psychiatric, Hyde et al., 2016, Okbay et al., 2016), but the findings are generally inconsistent. Although some of the inconsistent findings were probably due to the limited power of the original studies (Flint and Kendler, 2014), there may also be intrinsic heterogeneity across depression definitions. This is further supported by the fact that studies show very different estimates of the narrow-sense heritability (*h*_*n* _^2^) for several depression definitions or related traits to MDD (*h*_*n* _^2^ = 37% (Sullivan et al., 2000)): perceived stress: 44% (Bogdan and Pizzagalli, 2009); nine depression definitions in women: 21%–45% (Kendler et al., 1992); depressive symptom scores in childhood: 79% (Thapar and Mcguffin, 1994) and depressive symptoms in an elderly population: 69% in women and 64% in men (McGue and Christensen, 2003). In fact, even for MDD, the genetic correlation of MDD phenotypes between independent datasets was relatively low compared with other psychiatric disorders (Gratten et al., 2014).

Because of the heterogeneity across depression definitions, there has been a long debate about the correct phenotype for depression genetic studies. Studies using clinically-assessed depression could provide findings that are directly informative for clinical application. However, the resources required for such data collection are generally very high (consortium, 2015). As an alternative, measuring self-reported depression requires fewer resources and this phenotype is rapidly becoming available for many population-based datasets (Okbay et al., 2016, Hyde et al., 2016). To date, the largest published GWAS of major depression has yielded 15 significant loci (7 loci before meta-analysis) for a self-reported clinical diagnosis (Hyde et al., 2016).

Given that important progress is being made from GWASs on different depression definitions, it becomes increasingly important to understand the similarities and dissimilarities across different definitions. Particularly, the difference in genetic and environmental loadings might underpin the inconsistent results from genetic studies across different depression definitions. Therefore, dissecting the phenotypic variance of each depression definition and understanding the similarities and dissimilarities between those phenotypes in the context of both genetic and common environmental components is particularly important for interpreting the results from published depression studies and for informing about genetically relevant depression phenotypes for future studies.

In this study we sought to partition the phenotypic variation of the diagnosed depression (MDD) and the self-declared depression (SDD) into its genetic and familial environment components using Linear Mixed Modeling (LMM). Specifically, we utilized data from Generation Scotland: Scottish Family Health Study (GS:SFHS), a large Scottish cohort with extensive family relationship information and genome-wide genotype data to answer two questions. First, when simultaneously considering multiple genetic effects and familial environmental effects in the model, what are major contributions to variation in MDD and SDD, respectively? Second, what is the contribution of each of the identified major contributing components to the overall correlation between MDD and SDD?

## 2 Materials and Methods

The Tayside Research Ethics Committee (reference 05/S1401/89) provided ethical approval for the study. In GS: SFHS, participants gave written consent, after having an opportunity to discuss the project, and before any data or samples were collected.

The details of their consent status are recorded in the study database. All consent forms and study protocols were approved by the Research Ethics Committee.

### 2.1 Datasets

Generation Scotland: Scottish Family Health Study (GS: SFHS) contains 21,387 subjects (N_male_ = 8772, N_female_ = 12,615; Age_mean_ = 47.2), who were recruited from the registers of collaborating general practices. At least one first-degree relative aged 18 or over was required to be identified for each participant (Smith et al., 2006). Genotyping data were generated using the Illumina Human OmniExpressExome -8- v1.0 array (Gunderson, 2009). Details of genotyping are described elsewhere (Smith et al., 2006). Population outlier individuals were removed from the sample (Amador et al., 2015). Quality control (QC) of genotyped SNPs used inclusion thresholds: missing SNPs per individual ≤ 2%, SNP genotype call rate ≥ 98%, minor allele frequency (MAF) > 1% and Hardy-Weinberg equilibrium P value > 1 × 10^− 6^. In total, 561,125 genotyped autosomal SNPs passed QC criteria and were available for 19,994 participants (N_male_ = 8221, N_female_ = 11,773, Age_mean_ = 47.4).

#### 2.1.1 Phenotypes

Lifetime Diagnosis of MDD: The Structured Clinical Interview for DSM-IV(SCID) was used (First et al., 2012): participants who screened positive (21.7%) for the questions “Have you ever seen anybody for emotional or psychiatric problems? IF YES: What was that for? (What treatment(s) did you get? Any medications?) IF NO: Was there ever a time when you, or someone else, thought you should see someone because of the way you were feeling or acting” were invited to continue to an interview using the SCID modules for mood disorders (First et al., 2002). Participants who screened positive but refused to undergo the structured clinical interview (N = 507, 2.4%) and those with a diagnosis of bipolar disorder (N = 76) were excluded from the study. More details of phenotyping procedures are described elsewhere (Fernandez-Pujals et al., 2015).

Self-declared depression (SDD): the participants were invited to answer the following question “please mark an X in the box if you have been affected by depression”.

### 2.2 Partitioning the Phenotypic Variation

Based on the framework of the Genomic-relationship-matrix restricted maximum likelihood (GREML) method, Xia et al. (2016) developed a method to estimate *h*_*g*_^2^  (proportion of additive genetic variance contributed by common genetic variants over the total phenotypic variance, namely SNP heritability), *h*_*p*_^2^ (representing the additional additive genetic effect contributed by pedigree associated variation), *h*_*n*_^2^ (proportion of the total additive genetic variance over the total phenotypic variance, namely narrow-sense heritability) and a number of familial environmental components simultaneously (Xia et al., 2016). This was performed by fitting variance-covariance matrices representing common genetic effects, pedigree-related-genetic effects, and current and past family environmental effects simultaneously in the mixed linear model(Xia et al., 2016), building on previous work by Zaitlen et al. (Zaitlen et al., 2013). This approach enables estimation of the contribution of each genetic and family environmental component and here we applied it to MDD and SDD.

**Fig. 1:**
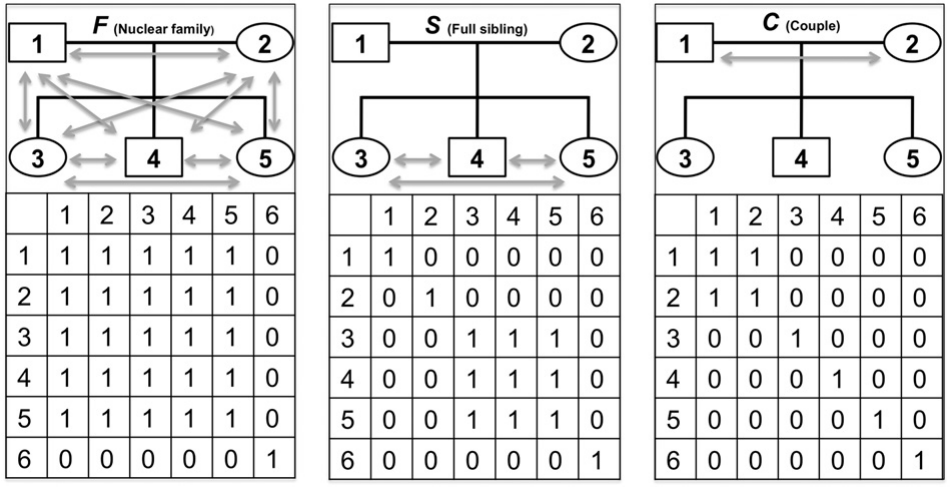
The design of the environment relationship matrices.

In detail, for each trait, two genomic relationship matrices, ***G*** (genomic relationship matrix) and ***K*** (kinship matrix created by modifying ***G*** using a threshold of 0.05 for pairwise relatedness) (Xia et al., 2016, Zaitlen et al., 2013)_,_ and three environment relationship matrices, ***F*** (environmental matrix representing nuclear-family-member relationships), ***S*** (environmental matrix representing full-siblings relationships) and ***C*** (environmental matrix representing couple relationships) (Fig. 1, Text s1) (Xia et al., 2016), were fitted separately or simultaneously in LMM (Text s2). The corresponding variance components *h*_*g*_^2^ (common-variant-associated genetic effect, represented in ***G***), *h*_*p*_^2^ (additional genetic effect from pedigree, represented in ***K***), *e*_*f*_^2^  (environmental effect from nuclear family, represented in ***F***)_,_*e*_*s*_^2^ (environmental effect from full sibling relationship, represented in ***S***)  and *e*_*c*_^2 ^ (environmental effect from couple relationship, represented in ***C***) were estimated in LMM and their significance was tested using LRT and Wald tests (Text s2). The estimates on the observed scale were transformed to the liability scale using the population prevalence of each trait (MDD: 0.13; SDD: 0.09). Age, age^2^, sex and the top 20 Principal components were included in the LMM as fixed effects. To simplify the model description, the following codes were used to represent the matrices fitted in the models: -e.g. ‘***GKFSC***’ was the full model which fits all five matrices as random effects simultaneously, and ‘***GKC***’ represents the model where the genomic relationship matrix, the kinship relationship matrix and the environmental matrix representing couple relationships were fitted simultaneously.

#### 2.2.1 Stepwise Model Selection for Identifying Major Contributing Components.

##### 2.2.1.1 Backward Stepwise Selection (Xia et al., 2016)

The selection started with the full model ‘***GKFSC***’. LRT and Wald tests were conducted for each variance component. A variance component was removed from the model if (1) it failed to obtain significance (at the 5% level) in both tests and (2) among the variance components satisfying (1), it had the highest *P* value in the Wald test. This process was performed repeatedly until all the remaining components obtained significance in at least one test.

##### 2.2.1.2 Forward Stepwise Selection

To check for the agreement of different model selection strategies, we additionally implemented a forward stepwise regression approach for model selection. In this case the selection started with a null model (without fitting any matrices). Matrices were then added into the model one at a time. LRT and Wald tests were conducted for each variance component. A variance component was added if (1) it obtained significance (at the 5% level) in both tests, (2) the addition of this component did not lead to the variance components already in the model becoming non-significant in both tests and (3) among the variance components satisfying both (1) and (2), it had the lowest P value in the Wald test. This process was repeated until no more variance components satisfied criteria (1), (2) and (3).

#### 2.2.2 Bivariate GREML Analysis for MDD and SDD

Using the selected model (***GKC***) obtained from the above analyses, we then estimated the relative contributions of each major contributing genetic and environmental component to the correlation between MDD and SDD. We used the GCTA-bivariate GREML analysis (Yang et al., 2011, Lee et al., 2012) to estimate the correlation between the two traits for the common-variant-associated genetic component, the pedigree-associated genetic component and the shared couple environment component simultaneously. Each estimated correlation was tested to determine the significance of its difference from both 0 and 1 by Log-likelihood test (LRT) and Wald test.

## 3 Results

In GS:SFHS, among the 19,994 participants with genome-wide genotyping data, we recognized 1742 pairs of couples, 8458 pairs of full siblings and 20,019 pairs of members living in the same nuclear family. In this dataset, 99.5% (19,896/19,994) of participants have MDD diagnosis information (2659 MDD cases and 17,237 controls) and 98% (19,603/19,994) of participants have answered the question for self-reported depression (1940 SDD cases and 17,663 controls) (the cross tabulation of MDD and SDD is given in Table s1).

### 3.1 The Full Model Partitions Phenotypic Variation into Genetic and Familial Environmental Components

**Fig. 2:**
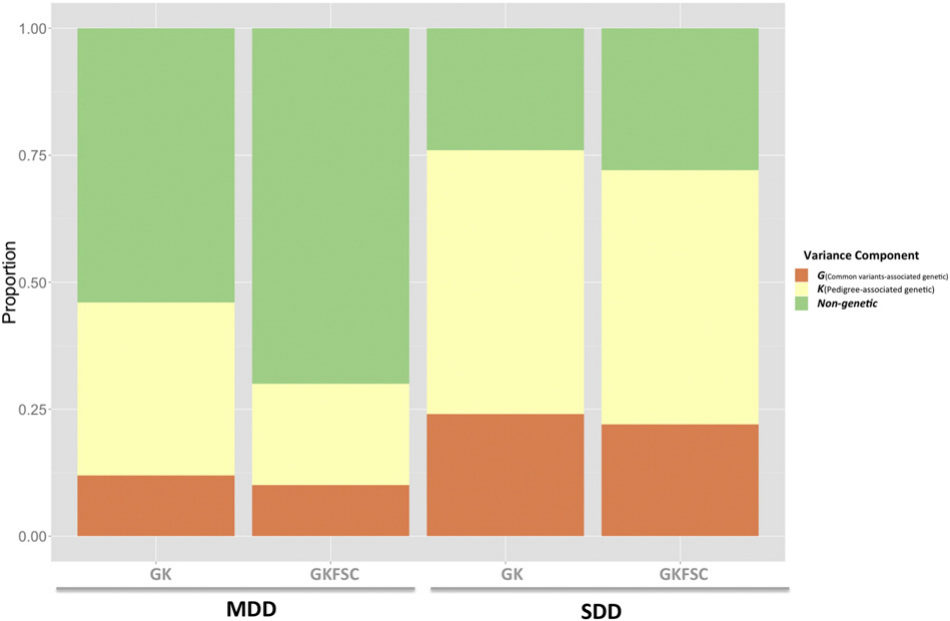
Comparison of the proportion of variance explained by the common-variants- and pedigree- associated genetic components estimated in the model that only account for the two genetic components (the ***GK*** model) and in the full model that accounts for two genetics and three shared-environmental effects (the ***GKFSC*** model). SDD: self declared depression, MDD: major depressive disorder.

A full model was first utilized to partition the phenotypic variation of each trait into five potential sources of influence: the additive genetic effect contributed by common variants (*h*_*g*_^2^), the additional additive genetic effect associated with pedigree (*h*_*p*_^2^), the environmental effect shared between all members of a nuclear family (*e*_*f*_^2^), the environmental effect shared between members of a couple (*e*_*c*_^2^) and the environmental effect shared between full siblings (*e*_*s*_^2^) (Fig. 1). The results are presented in Table 1. For MDD, 10% (se = 5%) of the phenotypic variance is attributable to the common genetic variants (*h*_*g*_^2^) and 20% (se = 12%) is to the additional genetic variation associated with pedigree (*h*_*p*_^2^). For SDD, 22% (se = 7%) of the phenotypic variance is attributable to the common genetic variants (*h*_*g*_^2^) and 50% (se = 15%) is attributable to the additional genetic variation associated with pedigree (*h*_*p*_^2^). The proportion of total additive genetic determinant (narrow-sense heritability: *h*_*n*_^2^ = *h*_*g*_^2^ +* h*_*p*_^2^) was 30% for MDD and 72% for SDD. For the three familial environmental components, the likelihood ratio test (LRT) for the couple-shared environmental effect was significant for SDD (17% (se = 10%)), but was not significant for MDD (although the estimated effect was non-zero: 3% (se = 9%)). The environmental effects shared between nuclear family members and full-siblings were not significant for either trait with the estimate of the sib effect being zero for both traits but for the family effect being non-zero for both traits. Compared with the a reduced model that does not account for environmental effects (the ***GK*** model), the full model obtains lower estimates of the genetic components for both traits, suggesting that the full model effectively reduced the confounding environmental effects when estimating the heritability (Fig. 2, Table 1).

### 3.2 Stepwise Model Selections Identify Major Genetic/Familial-Environment Contributors for MDD and SDD

As shown in previous work, the full model, although accounting for all of the five potentially influential effects, may have difficulty in separating major contributors to depression from minor contributors because of correlated effects (Xia et al., 2016). In order to address this problem (Xia et al., 2016), we applied stepwise model selection (Xia et al., 2016) to identify major contributors to variation in the two depression traits.

**Fig. 3:**
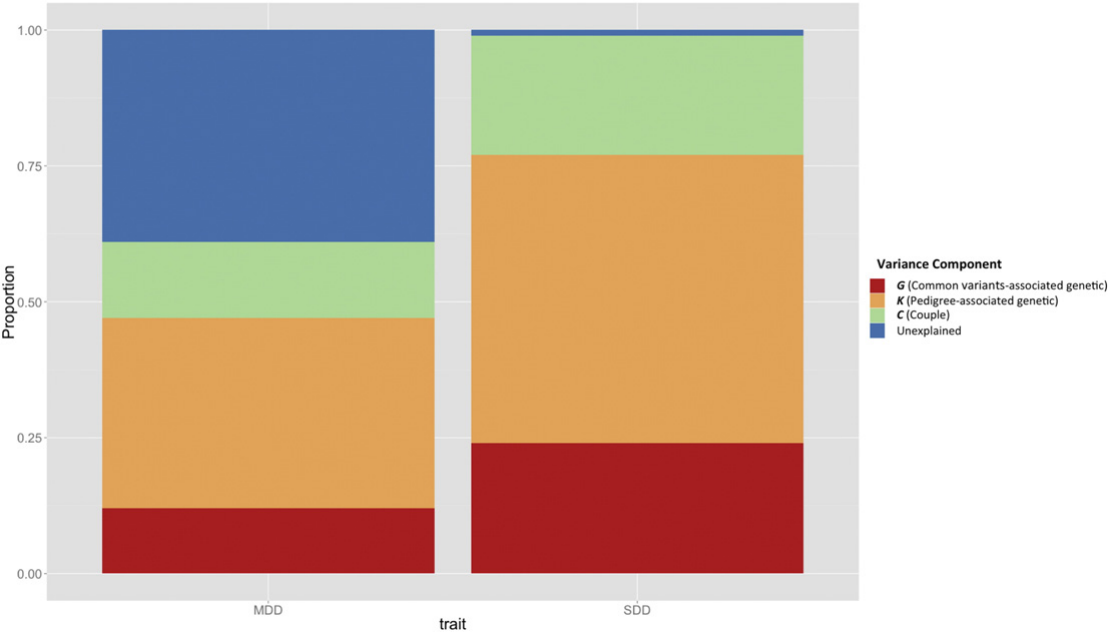
Sources of phenotypic variance and the proportion of variance they explained in the most parsimonious model (***GKC***) for both SDD and MDD. SDD: self declared depression, MDD: major depressive disorder.

Using forward stepwise selection, the common variant-associated-genetic effect, pedigree-associated-genetic effect and couple-shared environmental effects were retained in the final model for both MDD and SDD (the ***GKC*** model as shown in Table s2 and Table 1). Using backward stepwise selection, for MDD, only the common variant-associated-genetic component and shared-nuclear-family component were retained in the final model (the *GF* model as shown in Table s3a). For SDD, as for the forward stepwise approach, common variant-associated-genetic, pedigree-associated-genetic effect and couple-shared environmental effects were selected (the ***GKC*** model as shown in Table s3b). The relative contribution of each variance component to SDD and MDD in the ***GKC*** model is shown in Fig. 3.

### 3.3 Bivariate GREML Analyses Estimate the Genetic and the Environmental Correlations between MDD and SDD

Since the model that included common-variant- and pedigree- associated genetic and couple-shared environmental effects (the ***GKC*** model) was selected as the most parsimonious model in three out of the four model selections for the two traits. We further explored contributions of these three components to the correlation between MDD and SDD. The phenotypic correlation between MDD and SDD was 0.45 (Phi coefficient, P < 2.2 × 10^− 16^). The estimate of the common-variant-associated genetic correlation wsssas 1.00 (se = 0.20), this estimate being significantly different from 0 (*P*_*Wald_H*0*:*r_ _=_ _0_ < 1 × 10^− 5^, *P*_*lrt_H*0*:*r_ _=_ _0_ = 2.1 × 10^− 5^) and not significantly different from 1 (*P*_*Wald_H*0*:*r_ _= 1_ = 1, *P*_*lrt_H*0*:*r_ _=_ _1_ = 0.5). The estimate of the pedigree-associated genetic correlation was 0.57 (se = 0.08, *P*_*Wald_H*0*:*r_ _=_ _0_ < 1 × 10^− 5^, *P*_*Wald_H*0*:*r_ _=_ _1_ < 1 × 10^− 5^, *P*_*lrt_H*0*:*r_ _=_ _0_ = 7.7 × 10^− 10^, *P*_*lrt_H*0*:*r_ _=_ _1_ = 7.1 × 10^− 7^), and the estimate of the couple-shared environmental correlation was 0.52 (se = 0.22, *P*_*Wald_H*0*:*r_ _= 0_ = 0.016, *P*_*Wald_H*0*:*r_ _= 1_ = 0.03, *P*_*lrt_H*0*:*r_ _= 0_ = 0.07, *P*_*lrt_H*0*:*r_ _= 1_ = 0.06) (Table 2). Thus these two latter estimated correlations were significantly different from both zero and one in the Wald test.

## 4 Discussion

This variance-component analysis study assessed five genetic and familial environment risk contributions to MDD and SDD using GS:SFHS, a Scottish sample comprising close and distant relatives with genome-wide genotyping data. We showed that the common variant- and pedigree- associated genetics and the couple-shared environmental effects are major factors influencing liability to both clinically assessed and self-reported depression among the factors considered. The estimated correlation between SDD and MDD was very high (r = 1.00, se = 0.20) for common variant-associated genetic effects and lower for the pedigree-associated genetic (r = 0.57, se = 0.08) and couple-shared environmental effects (r = 0.52, se = 0.22).

The estimates of both common-variant-associated and pedigree-associated genetic effects are much lower for MDD (*h*_*g*_^2^ = 10%(5%), *h*_*p*_^2^ = 20%(12%)) than for SDD (*h*_*g*_^2^ = 22%(6%), *h*_*p*_^2^ = 50%(15%)), suggesting a difference between clinical and self-reported depression definitions. MDD is diagnosed through clinical questionnaire, whereas SDD reflects both depression status and the participants' self-awareness, this difference may underlie the higher heritability in SDD. The point estimate of SNP heritability *h*_*g*_^2^ for MDD (*h*_*g*_^2^ = 10%(5%)) is lower than that from a mega-analysis of 9 cohorts of European ancestry (21%(2%)) (Lee et al., 2013). This may be due to the intrinsic heterogeneity of MDD (Major Depressive Disorder Working Group of the Psychiatric et al., 2013). The pedigree-associated genetic component *h*_*p*_^2^ measures the additional genetic effects co-segregating in the pedigree (i.e. those not associated with common genetic variants), such as the effect of rare and structural variants. Using the full model which partitions the narrow sense heritability *h*_*n*_^2^ into the common-variant-associated component *h*_*g*_^2^ and the pedigree-associated component *h*_*p*_^2^,and accounts for multiple familial environmental effects simultaneously, the estimated *h*_*g*_^2^ accounted for around one third of *h*_*n*_^2^ in both MDD and SDD. This is lower than previous estimates for other complex traits (excluding depression) that suggest that > 50% of *h*_*n*_^2^ is accounted by *h*_*g*_^2^ (Zaitlen et al., 2013, Xia et al., 2016). Here *h*_*p*_^2^ accounted for more than two thirds of *h*_*n*_^2^, suggesting an important role for rare and structural variants in both clinical- and self-declared depression. The full model included a number of correlated matrices, potentially impeding model-fitting and their estimation (the discussion for collinearity is Text s3, Tables s4 and s5). A greater discriminating power may however have be achievable with larger sample sizes (Xia et al., 2016). In addition, the power to detect the effect might vary among tested components, therefore a caution should be made when compare the estimates of those components.

Stepwise model selections suggested that the two genetic effects and the couple-shared environmental effects were the most significant contributors to risk of depression among the factors considered (***GKC*** model was selected by both forward and backward selections for SDD and backward selection for MDD), although there is inconsistency in the results between the backward and the forward selections for MDD. This is probably due to the high correlation between ERM_family_ and the combined ERM_couple_ and GRM_kin_ (Xia et al., 2016). Simulation analysis of the backward selection method suggested that although it successfully selected the appropriate model with all major components of simulated phenotype in > 80% of cases, in 20% of cases the model either selected the ***F*** component when the true components were ***C*** plus ***K*** or the model selected ***C*** plus ***K*** but the true component was ***F*** (Xia et al., 2016)_._ Given that the pedigree-associated genetic component (**K**), a component that has been shown to be a major contributor of the genetic effect by the **GK** and the full model **GKFSC** in both MDD and SDD, was excluded by backward selection for MDD, it is likely we met the same problem as observed in simulation analysis by Xia et al. and the selected **GF** model is unlikely to contain all major resources of variation for MDD. As shown in simulation analysis, we expect that a larger sample size may provide sufficient power for a higher accuracy and stability of backward model selection (Xia et al., 2016). In addition, the estimates in the full model for MDD and SDD also suggest that components omitted from ***GKC*** model are likely to contribute only small amounts of variance, therefore ***GKC*** model was chosen as the best model for both traits in GS:SFHS. The significant contributions from the ***G***, ***K*** and ***C*** should be further replicated in independent samples.

In total, the selected ***GKC*** model explained 60% (se = 8%) and 98% (se = 9%) of the phenotypic variation in MDD and SDD, respectively (Fig. 3). Strikingly, the effect of the shared couple environment contributed as much as 14% (se = 7%) and 22% (se = 7%) of the phenotypic variance in MDD and SDD respectively. For GS:SFHS, an adult cohort with a minimum participant's age of 18, the couple effect reflects the current family environment shared between couples, which is contrast to the full sibling effect which reflects the influence of earlier shared environments. The role of the couple effect has previously been suggested in a Finnish study which showed that the partner's MDD status was associated with the MDD risk in non-psychiatric subjects (Joutsenniemi et al., 2011). Our results provided additional evidence for couple-associated effect and indicated that the magnitude of this effect is likely to be high. These results also suggest that the inclusion of partners in genetic studies of depression, whilst logistically attractive, might introduce confounding if additional adjustment is not made. It may be helpful for future genetic studies to be aware of these potential biases and to either avoid the recruitment of couples, or model their effects appropriately. In clinical practice, the mental health status of the spouse should also been considered as an additional indication of risk.

The couple-shared environmental effect detected in depression traits could be confounded by the non-random mating in those phenotypes. For example, assortative mating has been observed in multiple psychiatric disorders including depression. For SDD, ***G***, ***K*** and ***C*** explained a very high proportion of phenotypic variance. However assortative mating may also contribute to the fact that the total variance explained is so high. When assortative mating exists in the trait of interest, *h*_*g*_^2^, *h*_*n*_^2^ and *e*_*c*_^2^ estimates may be biased if this effect is not accounted for appropriately (Keller et al., 2013, Xia et al., 2016). The magnitude of this bias in heritability estimates, however, is likely to be small for diseases with high prevalence, such as MDD (Peyrot et al., 2016). On the other hand, ignoring the couple-shared environment effect in the study of assortative mating may also lead to inaccurate measurement of the degree of assortative mating. In GS:SFHS we did not detect significant genome-wide genotype-level assortative mating (Text s4, Figure s1) although the power of this test is very limited. The investigation of the genetic assortative mating in MDD- and SDD- associated loci has been impeded by the limited knowledge of the location of the loci that affect the trait (Charley Xia et al., personal communication). Further studies providing the age of marriage with large enough sample sizes to separate young and old couples would facilitate the discrimination of assortative mating from the effects of couple-shared environment.

Finally, we estimated the genetic and environment correlation between MDD and SDD. The results revealed a very high correlation in the common-variant-associated genetic component, a moderate correlation in the pedigree-associated genetic component and a less significant correlation in the couple-shared environment component. This suggests that there are strong genetic similarities between the depression phenotypes for common variants. In contrast, pedigree-associated genetic variation and shared environment may underpin important differences between these traits. This has important implications for the design of future molecular studies of depression in which SDD may be a good proxy phenotype for MDD in studies seeking to identify common risk variants. However, for family-based studies where the targeted genetic effect is from rare variants, the lower genetic correlation may impede the use of SDD as a proxy for MDD. The estimate of the correlation of the couple-shared environmental component has a wide confidence interval, which is likely due to the relatively limited number of couples in GS:SFHS. Therefore, replication of these findings in independent depression studies is indicated. In addition, since participants have multiple records (MDD and SDD), there might be other shared common environmental effects that are not specifically tested in current model but could be partitioned as one of the components already tested in the model (e.g. the correlation for ***G***, ***K*** or ***C*** could be inflated). Study designs using two independent datasets to infer the genetic correlation (such as LD-score regression) can be free of such shared environmental confounding effects.

## 5 Conclusions

This study showed that the common-variant- and pedigree-associated genetics and the couple-shared environmental effects are major factors influencing liability to depression. This suggests that the depression status of a spouse should be also treated as an indication of risk of depression. A high correlation of the common-variant-associated genetic component between SDD and MDD suggests that SDD could provide a scalable alternative to MDD in studies targeting common variants.

## Funding Sources

This work is supported by the Wellcome Trust through a Strategic Award, reference 104036/Z/14/Z. GS:SFHS was funded by a grant from the Scottish Government Health Department, Chief Scientist Office, number CZD/16/6. The authors acknowledge with gratitude the financial support received for this work from the Dr. Mortimer and Theresa Sackler Foundation. PAT, DJP and AMM are members of The University of Edinburgh Centre for Cognitive Ageing and Cognitive Epidemiology, part of the cross council Lifelong Health and Wellbeing Initiative (MR/K026992/1). Funding from the Biotechnology and Biological Sciences Research Council (BBSRC) and Medical Research Council (MRC) is gratefully acknowledged by PN and CSH (BB/J004235/1). DJM is an NRS Fellow, funded by the CSO.

## Conflict of Interest

AMM has previously received grant support from Pfizer, Lilly and Janssen. These studies are not connected to the current investigation. Other authors have no conflicts of interest.

## Authors' Contributions

Conceived and designed the experiments: YNZ AMM CSH PN DJP SPH BHS LJH AC. Performed the experiments: CH AC RN DJM AMFP. Analyzed the data: YNZ. Contributed reagents/materials/analysis tools: CX CSH PN YNZ AMM CA AMFP TKC. Collecting samples: BHS LJH SP DJM DJP AMM CSH. Wrote the paper: YNZ CSH AMM PN CA TKC JDH PAT and all the other authors. Raised funding: AMM CSH DJP.

## Figures and Tables

**Table 1:** Variance component analyses results for MDD and SDD.

			***G*** (Common variants -associated genetic)	***K*** (Pedigree-associated genetic)	***F*** (Nuclear family)	***S*** (Full sibling)	C (Couple)
Trait	Description Model		*h*_*g*_^2^(se)	*h*_*p*_^2^(se)	*e*_*f*_^2^(se)	*e*_*s*_^2^(se)	*e*_*c*_^2^(se)
MDD	Genetics only	***GK***	0.12(0.05)	0.34(0.06)			
	Full	***GKFSC***	0.10(0.05)	0.20(0.12)	0.09(0.06)^ns^	0.00(0.04)^ns^	0.03(0.09)^ns^
	Forward selection	***GKC***	0.12(0.05)	0.35(0.06)			0.14(0.07)
	Backward selection	***GF***	0.15(0.04)		0.16(0.03)		
SDD	Genetics only	***GK***	0.24(0.06)	0.52(0.07)			
	Full	***GKFSC***	0.22(0.06)	0.50(0.15)	0.04(0.07)^ns^	0.00(0.04)^ns^	0.17(0.10)
	Forward selection	***GKC***	0.24(0.06)	0.53(0.07)			0.22(0.07)
	Backward selection	***GKC***	0.24(0.06)	0.53(0.07)			0.22(0.07)

Variance component analyses were performed on MDD and SDD using the genetic model (***GK***), the model accounting for all of the two genetic and three familial environmental effects (the full model ***GKFSC***), and the models selected by forward or backward selection. NS: the variance component was not significant in LRT test.

**Table 2:** Bivariate GCTA-GREML estimates of the correlation of each variance component between SDD and MDD using ***GKC*** model.

	Test	MDD	SDD
*G* (Common variants-associated genetic)	*h*_*g*_^2^	0.10(0.05)	0·24(0.06)
	*r*_*g*_	1.00(0.20)	
*K* (Pedigree-associated genetic)	*h*_*p*_^2^	0.36(0.06)	0.52(0.07)
	*r*_*p*_	0.57(0.08)	
*C* (Couple)	*e*_*c*_^2^	0.13(0.07)	0·22(0.07)
	*r*_*c*_	0.52(0.22)	

## Appendix A Supplementary data

Supplementary material Image 1
