# Supplementary material for: Shared Genetics and Couple-Associated Environment Are Major Contributors to the Risk of Both Clinical and Self-Declared Depression
Source: eBioMedicine. 2016 Nov 4;14:161–7. doi: 10.1016/j.ebiom.2016.11.003 (PMC5161419; doi:10.1016/j.ebiom.2016.11.003)
Supplement: Supplementary file 1 — Supplementary material [file mmc1.docx]

**Shared genetics and couple-associated environment are major contributors to the risk of both clinical and self-declared depression**

***Supplementary Information***

Text s1 Constructing variation-covariance matrices representing different source of variation

Text s2 Estimating the phenotypic variance explained by different source of variation.

Text s3 Test for collinearity of the components estimated in the full and ***GKC*** models for MDD and SDD.

Text s4 Identity By States (IBS) of couples and random-grouped-and-distant-related cross sex pairs

Table s1. The number of participants in GS:SFHS with SDD or MDD.

Table s2. Forward stepwise model selection for a: MDD and b: SDD.

Table s3. Backward stepwise model selection for a: MDD and b: SDD.

Table s4. Correlation of predicted random effects of the five components in the full model. a: MDD. b: SDD.

Table s5. VIF of predicted random effects of the components in the full and ***GKC*** model.

Figure s1. The distributions of IBSs of couples and randomly-grouped-and-distant-related cross sex pairs.

**Text s1 Constructing variation-covariance matrices representing different source of variation**

***G*** *matrix:* Genomic relationship matrix. The genetic relationships between individuals were estimated by the formula:

$$Ajk=\frac{1}{N}\sum_{i=1}^{N} \frac{(xij-2pi)(xik-2pi)}{2pi(1-pi)}$$

where i is a SNP, x is the allelic dosage of the minor allele for individual j or k at i. p_i_ is the minor allele frequency of i , N is the total number of SNPs. This matrix was created in GCTA([Yang *et al.*, 2011](#_ENREF_3)).

***K*** *matrix*: Kinship relationship matrix. ***K*** matrix was created by modifying ***G*** matrix. We set values of the relationship coefficient less than or equal to 0.05 in ***G*** matrix to 0. The threshold of 0.05 was applied as it separates closely and distantly related individuals([Zaitlen *et al.*, 2013](#_ENREF_4)).

*Environment relationship matrices*

Three matrices were designed for representing shared environmental effects from different familial relationships: .***F*** (environmental matrix representing nuclear-family-members living in the same household), ***S*** (environmental matrix representing full-siblings relationships) and ***C*** (environmental matrix representing couple relationships). To create each matrix, we performed the following steps: (1) create an N by N matrix and set all entries to 0. (2) set diagonal entries to 1. (3) for the off-diagonal entries, an entry would be set to 1 if the two individuals share the family environment of interest(Figure 1,Table s2).

**Text s2 Estimating the phenotypic variance explained by different source of variation.**

The genomic and environment relationship matrices described above were selectively jointly fitted in a LMM implemented in GCTA. The models analyzed included all the fixed effect and the subsets of random effects in the full model:

***Y = Xb + g_g_ + g_kin_ + e_f_ + e_s_ + e_c_ + ε***

Where Y was a vector of MDD binary phenotypes, b was a vector of covariates fitted as fixed effects (i.e., age, age2, sex, 20 principal components derived from the genome-wide genomic relationship matrix). g_g_ and g_kin_ were the random genetic effects from the SNPs and the extra random genetic effect from the pedigree, respectively, with g_g_ ~ *N* (0, ***G***$\boldsymbol{\sigma}_{\boldsymbol{g}}^{\boldsymbol{2}}$) and g_kin_ ~ *N* (0, ***K***$\boldsymbol{\sigma}_{\boldsymbol{kin}}^{\boldsymbol{2}}$). e_f_, e_s_, e_c_ represent the random environmental effects shared by nuclear family members, full-siblings and couples, respectively. To simplify the model description, the following codes were used to represent the matrices fitted in the models: -e.g. ‘***GKFSC***’ was the full model which fits all five matrices as random effects simultaneously, and ‘***GKC***’ represents the model where the genomic relationship matrix, the kinship matrix and the environmental matrix representing couple relationships were simultaneously fitted. The proportion of variance explained by individual component was estimated using REML and tested using Log-likelihood Ratio Test (LRT) in GCTA (Population prevalence used in transferring estimates to liability scale: 0.13 for MDD and 0.09 for SDD)([Yang *et al.*, 2011](#_ENREF_3)).

**Text s3 Test for collinearity of the components estimated in the full and *GKC* models for MDD and SDD.**

To test for the collinearity of variance components, we predicted the random effects (in animal models, this is called ‘breeding value’) of each component in each individual in the full and ***GKC*** models for MDD and SDD by best linear unbiased prediction (BLUP) in GCTA([Yang *et al.*, 2011](#_ENREF_3)). The correlation between the predicted random effects of the components in the full model were generally high for both MDD and SDD (Table s4). The collinearity was tested by calculating the variance inflation factor (VIF) for the predicted random effect of each component in the full and the ***GKC*** models for both MDD and SDD. AVIF of less than 10 is indicative of inconsequential collinearity**(**[**Hair *et al.*, 1995**](#_ENREF_1)**).** The results were shown in Table s5. The VIF scores are higher in the full model compared with the selected model (***GKC***) by stepwise model selection. The ***G*** and ***K*** components have higher VIFs in the full model and that is likely to be contributed by each other and the three familial environmental components. The three environmental components were less impacted by each other or the two genetic components. The pattern of the collinearity among those components is similar in models for MDD and SDD. Whilst these results suggested that the collinearity existed in the full model and the stepwise model selection effectively reduced the collinearity between components, it is worth mentioning that the discrimination power for those components could be increased when the sample size is increased (the colinearity is not associated with the sample size), as shown in previous study([Xia *et al.*, 2016](#_ENREF_2)).

**Text s4 Identity By States (IBSs) of couples and randomly-grouped-and-distant-related cross sex pairs**

Identity By States (IBSs) were extracted from the GRM (the ***G*** matrix) created by GCTA. Randomly-grouped-and-distant-related cross sex pairs were generated based on an unrelated dataset of GS:SFHS. This unrelated dataset includes 7388 distant related participants and was generated in GCTA by removing one of each pair of individuals with an estimated relatedness larger than 0.025 while maximizing the sample size. This generated 8805300 randomly-grouped-and-distant-related cross sex pairs. Among the 1742 pairs of couples, nine of them had an IBS larger than 0.025 and were removed from this analysis. The T-test (two-tailed) was conducted to compare the IBSs of couple pairs and the IBS of randomly-grouped-and-distant-related cross sex pairs. The result showed that the mean of IBSs of couple pairs was not significantly different from the IBS of randomly-grouped-and-distant-related cross sex pair (P=0.09, Mean_cross-sex_=-6.5E-05, Mean_couple_=1.1E-04). The distributions of IBSs of the two groups were shown in Figure s1. Therefore In current study we didn’t observe a significant genome-wide genotype level assortative mating in GS:SFHS.

|  | SDD Controls | SDD-Cases | Unknown |
| --- | --- | --- | --- |
| MDD Controls | 16157 | 746 | 334 |
| MDD Cases | 1462 | 1146 | 51 |
| Unknown | 44 | 48 | 6 |

**Table s1. The number of participants in GS:SFHS with SDD or MDD.**

**a**

| Variance component | 1^st^ round | | | 2^nd^ round | | | 3^rd^ round | | | 4^th^ round | | |
| --- | --- | --- | --- | --- | --- | --- | --- | --- | --- | --- | --- | --- |
|  | P_lrt_ | estimate | se | P_lrt_ | estimate | se | P_lrt_ | estimate | se | P_lrt_ | estimate | se |
| ***K (Pedigree genetics)*** | 1.57E-34 | 0.46 | 0.04 |  |  |  |  |  |  |  |  |  |
| ***G (Common variants genetics)*** | 7.24E-29 | 0.33 | 0.03 | 5.58E-03 | 0.12 | 0.05 |  |  |  |  |  |  |
| ***C (Couple)*** | 1.83E-02 | 0.16 | 0.07 | 3.45E-02 | 0.14 | 0.07 | 3.27E-02 | 0.14 | 0.07 |  |  |  |
| ***F (Nuclear family)*** | 7.81E-33 | 0.26 | 0.02 | 2.10E-02 | 0.10 | 0.04 | 2.11E-02 | 0.10 | 0.04 | 1.21E-01 | 0.07 | 0.06 |
| ***S (Full sibling)*** | 4.51E-14 | 0.20 | 0.03 | 5.00E-01 | 0.00 | 0.03 | 5.00E-01 | 0.00 | 0.03 | 5.00E-01 | 0.00 | 0.04 |

**b**

| Variance component | 1^st^ round | | | 2^nd^ round | | | 3^rd^ round | | | 4^th^ round | | |
| --- | --- | --- | --- | --- | --- | --- | --- | --- | --- | --- | --- | --- |
|  | P | estimate | se | P | estimate | se | P | estimate | se | P | estimate | se |
| ***K (Pedigree genetics)*** | 3.38E-72 | 0.76 | 0.05 |  |  |  |  |  |  |  |  |  |
| ***G (Common variants genetics)*** | 2.81E-64 | 0.57 | 0.04 | 8.61E-06 | 0.24 | 0.06 |  |  |  |  |  |  |
| ***C (Couple)*** | 1.08E-03 | 0.25 | 0.07 | 2.35E-03 | 0.22 | 0.07 | 2.06E-03 | 0.22 | 0.07 |  |  |  |
| ***F (Nuclear family)*** | 5.46E-69 | 0.42 | 0.03 | 2.50E-02 | 0.10 | 0.05 | 2.56E-02 | 0.10 | 0.05 | 4.69E-01 | 0.01 | 0.07 |
| ***S (Full sibling)*** | 4.02E-30 | 0.34 | 0.03 | 5.00E-01 | 0.00 | 0.04 | 5.00E-01 | 0.00 | 0.04 | 5.00E-01 | 0.00 | 0.04 |

**Table s2. Forward stepwise model selection for a: MDD and a: SDD.** The selection started with a model without any matrix fitted. The variance component highlighted in colour: the component was added after the selection in current round. P_lrt_ : . the P value of LRT by comparing a model with the target variance component fitted against a reduced model without it fitted. Estimate: the estimate of the variance explained by the target component. The grey words: in MDD, the ***F*** matrix was not added in the 3^rd^ round, as adding it leads to the ***K*** component (added in the 1^st^ round) becoming insignificant.

| Variance component | 1^st^ round(Full) | | | 2^nd^ round | | | 3^rd^ round | | | 4^th^ round | | |
| --- | --- | --- | --- | --- | --- | --- | --- | --- | --- | --- | --- | --- |
|  | P_lrt_ | estimate | se | P_lrt_ | estimate | se | P_lrt_ | estimate | se | P_lrt_ | estimate | se |
| ***G (Common variants genetics)*** | 5.36E-03 | 0.10 | 0.05 | 5.45E-03 | 0.12 | 0.05 | 5.60E-03 | 0.12 | 0.05 | 3.15e-05 | 0.15 | 0.04 |
| ***F (Nuclear family)*** | 1.20E-01 | 0.09 | 0.06 | 1.21E-01 | 0.07 | 0.06 | 2.11E-02 | 0.10 | 0.04 | 2.56E-09 | 0.16 | 0.03 |
| ***K (Pedigree genetics)*** | 2.56E-02 | 0.20 | 0.12 | 4.14E-02 | 0.22 | 0.12 | 6.35E-02 | 0.16 | 0.10 |  |  |  |
| ***C (Couple)*** | 2.47E-01 | 0.03 | 0.09 | 2.12E-01 | 0.08 | 0.09 |  |  |  |  |  |  |
| ***S (Full sibling)*** | 5.00E-01 | 0.00 | 0.04 |  |  |  |  |  |  |  |  |  |

**a**

**b**

| Variance component | 1^st^ round(Full) | | | 2^nd^ round | | | 3^nd^ round | | |
| --- | --- | --- | --- | --- | --- | --- | --- | --- | --- |
|  | P_lrt_ | estimate | se | P_lrt_ | estimate | se | P_lrt_ | estimate | se |
| ***K (Pedigree genetics)*** | 2.02E-04 | 0.50 | 0.15 | 2.52E-04 | 0.52 | 0.15 | 2.97E-14 | 0.53 | 0.07 |
| ***G (Common variants genetics)*** | 7.53E-06 | 0.22 | 0.06 | 7.61E-06 | 0.24 | 0.06 | 7.57E-06 | 0.24 | 0.06 |
| ***C (Couple)*** | 1.98E-02 | 0.17 | 0.10 | 1.76E-02 | 0.22 | 0.10 | 2.06E-03 | 0.22 | 0.07 |
| ***F (Nuclear family)*** | 2.99E-01 | 0.04 | 0.07 | 4.69E-01 | 0.01 | 0.07 |  |  |  |
| ***S (Full sibling)*** | 5.00E-01 | 0.00 | 0.04 |  |  |  |  |  |  |

**Table s3. Backward stepwise model selection for a: MDD and b: SDD.** The selection started with the full model ‘***GKFSC***’. The variance component(s) highlighted in colour: the component remained after the selection in current round. P_lrt_ : . the P value of LRT by comparing a model with the target variance component fitted against a reduced model without it fitted. Estimate: the estimate of the variance explained by the target component.

|  | S | C | F | K | G |
| --- | --- | --- | --- | --- | --- |
| S | 1.00 | 0.67 | 0.77 | 0.84 | 0.76 |
| C | 0.67 | 1.00 | 0.58 | 0.76 | 0.68 |
| F | 0.77 | 0.58 | 1.00 | 0.88 | 0.79 |
| K | 0.84 | 0.76 | 0.88 | 1.00 | 0.90 |
| G | 0.76 | 0.68 | 0.79 | 0.90 | 1.00 |

|  | S | C | F | K | G |
| --- | --- | --- | --- | --- | --- |
| S | 1.00 | 0.65 | 0.76 | 0.83 | 0.74 |
| C | 0.65 | 1.00 | 0.56 | 0.74 | 0.67 |
| F | 0.76 | 0.56 | 1.00 | 0.87 | 0.78 |
| K | 0.83 | 0.74 | 0.87 | 1.00 | 0.90 |
| G | 0.74 | 0.67 | 0.78 | 0.90 | 1.00 |

**a**

**b**

**Table s4. Correlation of predicted random effects of the five components in the full model. a: MDD. b: SDD.**

| Trait | **Model** | ***G***  (Common variants  -associated genetic) | ***K***  (Pedigree-associated  genetic) | ***F***  (Nuclear family) | ***S***  (Full sibling) | C  (Couple) |
| --- | --- | --- | --- | --- | --- | --- |
| MDD | ***GKFSC*** | 17.23 | 19.88 | 3.83 | 2.81 | 2.44 |
|  | ***GKC*** | 3.13 | 1.83 |  |  | 2.27 |
| SDD | ***GKFSC*** | 25.60 | 20.76 | 4.68 | 1.71 | 2.68 |
|  | ***GKC*** | 1.89 | 1.73 |  |  | 1.53 |

**Table s5. VIF of predicteds random effects of the components in the full and *GKC* model.**


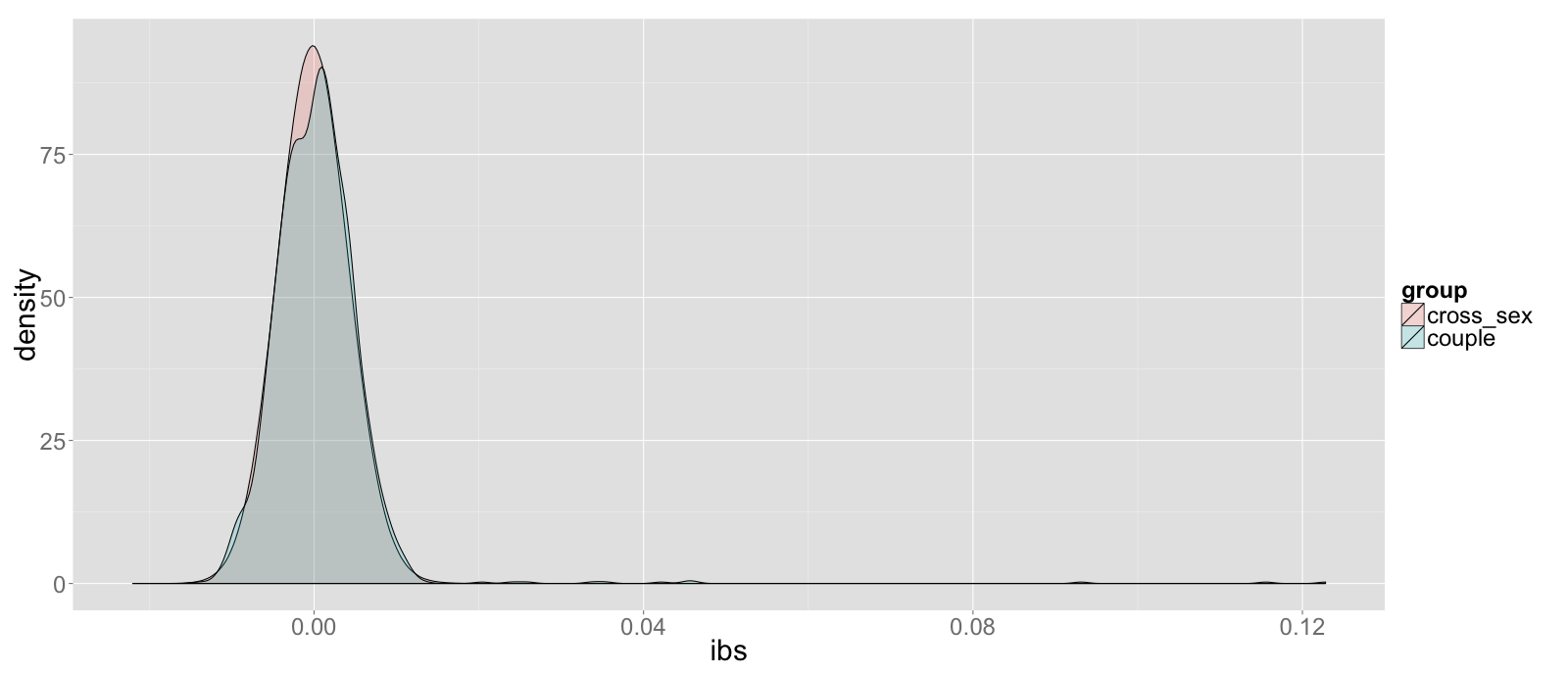


Figure s1. The distributions of IBSs of couples and randomly-grouped-and-distant-related cross sex pairs.

**References**

Hair, J. F., Black, B., Anderson, R. & Tatham, R. 1995. Multivariate Data Analysis: Text and Readings.

Xia, C., Amador, C., Huffman, J., Trochet, H., Campbell, A., Porteous, D., Hastie, N. D., Hayward, C., Vitart, V., Navarro, P., Haley, C. S. & Generation, S. 2016. Pedigree- and SNP-Associated Genetics and Recent Environment are the Major Contributors to Anthropometric and Cardiometabolic Trait Variation. *PLoS Genet,* 12**,** e1005804.

Yang, J., Lee, S. H., Goddard, M. E. & Visscher, P. M. 2011. GCTA: a tool for genome-wide complex trait analysis. *Am J Hum Genet,* 88**,** 76-82.

Zaitlen, N., Kraft, P., Patterson, N., Pasaniuc, B., Bhatia, G., Pollack, S. & Price, A. L. 2013. Using Extended Genealogy to Estimate Components of Heritability for 23 Quantitative and Dichotomous Traits. *Plos Genetics,* 9.
